# p53 activation and mitochondria-mediated pathway are involved during hanging death-induced neuronal cell apoptosis in dentate gyrus region of the rat brain

**DOI:** 10.1186/2193-1801-2-407

**Published:** 2013-08-27

**Authors:** Sabana Khatun, Shail K Chaube, Chandra N Bhattacharyya

**Affiliations:** Central Forensic Science Laboratory, Directorate of Forensic Science Services, Ministry of Home Affairs, Govt of India, 30 Gorachand Road, Kolkata, 700014 India; Cell Physiology Laboratory, Department of Zoology, Banaras Hindu University, Varanasi, 221005 India

**Keywords:** Forensic science, Hanging death, p53, Cytochrome c, Caspases activity, Apoptosis, Necrosis, Neuronal cells

## Abstract

The goal of this study was to understand the molecular event in the brain caused by hanging death (HD). Animals were subjected to either cervical dislocation (CD) or HD. Brain was collected at various times (0, 1, 3, 6 and 12 h) after death. Brain expression of p53 and Bax, cytochrome c concentration, caspases activity and DNA fragmentation were analyzed. Compared to that of CD, HD increased p53 and Bax proteins expressions, cytochrome c concentration, caspases activity and DNA fragmentation during the early period (0–6 h) of HD, whereas CD induced necrosis 3 h post- CD and thereafter. These data support that HD induces neuronal cell apoptosis, in part, through mitochondria-mediated pathways. These data also suggest that neuronal apoptosis could be a potential marker and an aid to forensic science of HD.

## Introduction

Hanging is one of the most common approaches to commit suicide in human (Emet et al. 2010) and generates permanent global brain ischemia by involving irreversible stoppage of blood supply to the brain (Akdemir and Ergungor 1994; Oechmichen and Meissner 2006; Yamasaki et al. 2007). Cessation or severe reduction of blood supply to the whole brain deprive brain tissue and neuronal cells from required oxygen and nutrients to sustain survival and generates global brain ischemia. Global brain ischemia induces neuronal cell apoptosis in most susceptible areas (Traystman 2003) including dentate gyrus (DG) region of the hippocampus (MacManus et al. 1993; Jung and Hong 2004; Lebesgue et al. 2009) possibly through the generation of reactive oxygen species (ROS) including hydrogen peroxide (H_2_O_2_) (Hyslop et al. 1995).

The overproduction of ROS from mitochondria induces p53 activation (Yu et al. 1999; Kunimatsu et al. 2011) and thereby neuronal cell apoptosis (Endo et al. 2006; Chan 2005). Studies suggest that Bax (a pro-apoptotic member of the bcl-2 gene family) is essential for p53-induced apoptosis (Xiang et al. 1998). The Bax gene contains p53 consensus sequences within its promoter and transcriptionally regulated by p53 (Miyashita et al. 1994; Miyashita and Reed 1995). The translocation of p53 to the mitochondria by alterations in the activity of Bax induces cytochrome c release to the cytosol of neuronal cells after global brain ischemia (Xiang et al. 1998; Sugawara et al. 1999; Cao et al. 2001; Mihara et al. 2003; Hong et al. 2010). The increased cytosolic cytochrome c level initiates apoptotic signals in neuronal cells (Endo et al. 2006) but the downstream pathway after global brain ischemia remains to be elucidated. However, few studies indicate that the cytochrome c binds to apoptotic factor-1 (Apaf-1) leading to the recruitment and activation of procaspase-9 (Krajewski et al. 1999; Mouw et al. 2002; Plesnila 2004). As a result, procaspase-9 auto processes and cleaves the procaspase-3 (Namura et al. 1998; Ferrer et al. 2003; Chan 2004). The activated procaspase-3 cleaves key structural and regulatory proteins and induces morphological apoptotic changes in neuronal cells (Namura et al. 1998; Sugawara et al. 2002,2004).

A possibility exist that similar to global brain ischemia induced due to various pathological and/or experimental conditions; hanging death (HD) may induce p53 activation and neuronal cell apoptosis through mitochondria-mediated pathway in DG region of the hippocampus. Although HD is one of the major clinically important forensic science issues, it is not possible to carry out studies about hanging on human subjects for ethical reasons. Animal models can be substituted for human experimentation (Boghossian et al. 2010). Models of rats hanged by the neck with a rope have already been developed for the induction of the expression of hypoxia-inducible factor −1 (HIF-1), a marker of hypoxia (Zhang et al. 2006). We have recently reported that HD induces generation of reactive oxygen species (ROS) and neuronal cell apoptosis in the dentate gyrus (DG) region of rat brain (Khatun et al. 2013) but the underlying mechanism during HD-induced neuronal cell apoptosis remains to be elucidated. Hence, present study was aimed to find out whether activation of p53 and mitochondria-mediated pathway are involved during HD-induced neuronal cell apoptosis. For this purpose, permanent global brain ischemia was induced by hanging in experimental rats after anaesthesia. The histology, p53 and Bax expressions, cytochrome c concentration, caspase-9 and caspase-3 activities and DNA fragmentation were analysed in neuronal cell of the DG region of rat brain.

## Materials and methods

### Chemicals and animal maintenance

All chemicals used in the present study were purchased from Sigma Chemical Co. (St. Louis, MO, USA), unless stated otherwise. Sexually mature male rats (60 days old; 150 gm ± 5 body weight; bw) of Charles-Foster strain were separated from existing colony of departmental animal facility and maintained in normal husbandry conditions with food and water ad libitum. All procedures confirmed to the stipulations of the Departmental animal ethical committee of Jadavpur University, Jadavpur, Kolkata, West Bengal and followed the guidelines for the care and use of laboratory animals (NIH Publication).

### Induction of permanent global brain ischemia

Experimental rats were exposed to anaesthesia to minimize the pain and then quickly hanged till death to induce permanent global brain ischemia. For this purpose, rats were exposed to light inhalation anaesthesia using anaesthetic grade ether in an air-tight glass jar for 2 min. The semiconscious rats were removed from the glass chamber and then hanged till death. The heart beat was felt by gentle keeping the thumb on the chest region and stoppage of heart beat was used as criteria to determine as 0 h time after death. The average time for HD after anaesthesia was 3.0 ± 1.0 min and total 60 rats were used to minimise the number of rats used in the present study. The rats were left in hanging position at room temperature and brain was isolated after various times (0,1,2,3,4,5,6,9 and 12 h) of HD to mimic the conditions of suicidal cases due to hanging in human. Another group of animals (control group) were exposed to light inhalation anaesthesia as described above and subjected to cervical dislocation (control). The rats were left at room temperature and brain was isolated after various times as described above for HD group. The brain was kept separately in sterile phosphate buffer saline (PBS; pH 7.4) solution and then processed for cellular and biochemical analyses. Based on the biochemical results obtained in the present study, we reduced number of time points and brain samples collected after 0,1,3,6 and 12 h of CD and HD were only processed for histology and immunohistochemistry.

### Histology of DG region of the hippocampus

For histology, brain from control and permanent global brain ischemia induced due to hanging (0,1,3,6 and 12 h) was removed and placed in buffered formaldehyde (3.7% in 10 mM PBS; pH 7.4) for 24 h, embedded in paraffin by the routine method, serially sectioned at 5 μm thickness, and stained in haematoxylin / eosin for microscopic observation. Experiment was repeated at least three times using brain sample of three independent animals for each time point and representative photographs at 400X magnification are shown in the result section.

### Immunocytochemistry for p53

Immunostaining for p53 was carried out using anti- p53 antibody following company (Santa Cruz Biotechnology Inc., CA, USA) manual protocol with some modifications. Briefly, sections of paraffin-embedded brain from control and permanent global brain ischemia induced due to hanging (0, 1, 3, 6 and 12 h) were deparaffinised in xyline overnight and then rehydrated with different graded ethanol. The slides were washed thrice with phosphate buffered saline (PBS) (0.01 M, pH 7.4) and then treated with 0.3% H_2_O_2_ in absolute methanol for 15 min to quench endogenous peroxidase activity. The slides were washed twice with PBS and then exposed to PBS containing 0.1% Triton X-100 to for permeabilization. Slides were then exposed to 100 μl of blocking buffer (0.5% BSA, 0.1% tween-20 in 100 ml PBS) at room temperature for 1 h and then incubated with an anti-p53 antibody (sc-6423; Santa Cruz Biotechnology), followed by SYBR green-conjugated anti-rabbit IgG antibody (Santa Cruz Biotechnology Inc., CA, USA) at a dilution of 1:1000 in PBS at room temperature for 1 h in a humidified chamber. At the end of the incubation period, slides were washed three times with PBS then mounted in fluorescence mounting medium. The mounted slides were photographed using Nikon inverted fluorescent microscope (TE-Eclipse 800; Tokyo, Japan). The experiment was repeated three times using brain sample of three independent animals for each time point and a representative photograph is shown in the result section. The p53 positive cells were counted stereologically within the DG region of the hippocampus at 400X magnification using brain sample of three independent animals.

### Immunocytochemistry for Bax

Immunostaining for Bax was carried out using anti- Bax antibody following company (Santa Cruz Biotechnology Inc., CA, USA) manual protocols. In brief, paraffin-embedded brain sections from control and permanent global brain ischemia induced due to hanging (0, 1, 3, 6 and 12 h) were treated as described above for the immunocytochemistry of p53. Slides were then incubated with an anti-Bax antibody (sc-6423; Santa Cruz Biotechnology), followed by SYBR green-conjugated anti-rabbit IgG antibody(Santa Cruz Biotechnology Inc., CA, USA) at a dilution of 1:1000 in PBS at room temperature for 1 h in a humidified chamber. At the end of the incubation period, slides were washed three times with PBS then mounted in fluorescence mounting medium. The mounted slides were photographed using Nikon inverted fluorescent microscope (TE-Eclipse 800; Tokyo, Japan). All samples (using brain sample of three independent animals for each time point) were run in the same assay to avoid inter-assay variation. The Bax positive cells were counted stereologically within the DG region of the hippocampus at 400X magnification using brain sample of three independent animals.

### Quantitative analysis of intracellular cytochrome c concentration

The cytochrome c concentration in brain homogenates were analyzed using cytochrome c ELISA kit purchased from R&D Systems MN, U.S.A. The brain was isolated from control and experimental animals, washed three times with PBS and homogenised in 10 ml of hypotonic lysis buffer (10 mM Tris–HCl,150 mM NaCl, 5 mM EDTA, 1% TritonX-100,0.1% SDS, 1 mM PMSF, 0.28 U Apoprotein, pH 8.0). The homogenates were centrifuged at 14000 × g at 4°C for 20 min and clear supernatant was immediately used for the quantitative estimation of cytochrome c by colorimetric assay as per company manual protocol. The optical density (OD) was determined using a microplate reader (Micro Scan MS5608A, ECIL, Hyderabad, India) set at 450 nm. All samples (3 independent samples for each time point) were run in the same assay to avoid inter-assay variation.

### Caspase activity assays

The intracellular caspase-3 and caspase-9 activities in brain homogenates were analyzed using colorimetric assay kits purchased from R&D Systems MN, USA. In brief, brain homogenates were prepared as described above for the measurement of cytochrome c concentration. A clear supernatant was removed from each sample and stored at -30°C until assay. Samples were quickly thawed and 50 μl of each sample (in triplicate) was loaded in 96 microwells plate. The 50 μl of 2X reaction buffer (buffer 9 for caspase-9 and buffer 3 for caspase-3 assay provided with the kit) was added in each well. The 5 μl of caspase substrate (LEHO-pNA, caspase-9 substrate; DEVD-pNA, caspase-3 substrate) was added to each well and then plates were incubated at 37°C for 2 h. At the end of incubation period, plates were read at 405 nm using microplate reader (Micro Scan MS5608A, ECIL, Hyderabad, India) for caspase-9 and caspase-3 activities. All samples (using brain sample of three independent animals for each time point) were run in one assay to avoid inter-assay variation and OD values are directly used to depict caspase-9 and caspase-3 activities.

### DNA fragmentation analysis

The DNA fragmentation was assessed by fluorescence microscopy using acridine orange/ethidium bromide (AO/EB) staining following the published protocol (Natesan et al. 2006). Briefly, sections of paraffin-embedded brain from control and permanent global brain ischemia induced due to hanging (0, 1, 3, 6 and 12 h) were deparaffinised in xylene overnight and then rehydrated with different graded ethanol. The slides were washed once with phosphate buffered saline (PBS) and then treated with 100 μL of 1:1 mixture of AO/EB solutions (4 μg/mL) for 2 min as per the published methods (Priyadarsini et al. 2010). The slides were washed with PBS and then photographed using Nikon inverted fluorescent microscope (TE-Eclipse 800; Tokyo, Japan). The viable cells, apoptotic cells and necrotic cells were identified depending on their color change due to binding of ethidium bromide to fragmented DNA as described earlier (Gohel et al. 1999). Normal cells with intact DNA had green fluorescence of AO. The extent of EB binding to fragmented DNA changes the color from green to yellow (for early apoptotic cells), orange color (late apoptotic cells) and bright red color (necrotic cell). Experiment was repeated three times using brain sample of three independent animals for each time point and representative photographs are shown in result section.

### Statistical analyses

Data are expressed as mean ± S.E. of mean (S.E.M.) of 3 independent animals. Data were analyzed either by One-Way or by Two-Way analysis of variance (ANOVA) followed by Tukey test using SPSS software, version 17.5 (SPSS, Inc. Chicago, IL, USA). A probability of p < 0.001 was considered to be statistically significant.

## Results

### HD induces morphological features characteristics of apoptosis

**Figure 1:**
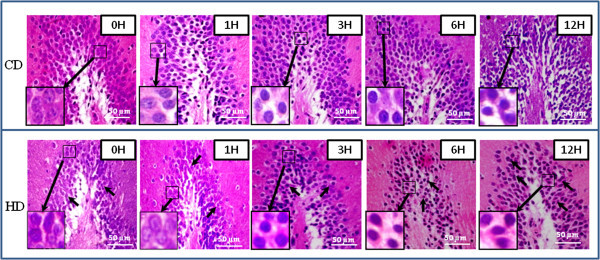
**Representative photographs showing HD-induced histological changes in DG region of hippocampus.** HD induced shrinkage and scattering of neuronal cells with gradual decrease in number during early period (Figs. HD, 1–6 H; arrows) as compare to their respective CD groups (Figs. CD, 1–6 H). CD induced disruption of histoarchitecture of DG region and increased necrotic changes after 6 h (CD, 12 H). Enlarge view of morphological changes in neuronal cells of the same figure (inset). Scale bar = 50 μM.

As shown in Figure 1, HD induced histological changes in DG region of hippocampus. Time-dependent changes in the histoarchitecture of the DG region were observed in both HD as well as CD groups. Permanent global brain ischemia generated due to HD gradually reduced total number of neuronal cells, and induced morphological apoptotic features such as shrinkage and scattering of neuronal cells in the DG region (Figs. HD, 1–12 H). On the other hand, CD group did not reduce the total number of cells but the histoarchitecture of DG region was gradually disrupted after 6 h of CD (Figs. CD, 1-12H).

### HD induces p53 activity

**Figure 2:**
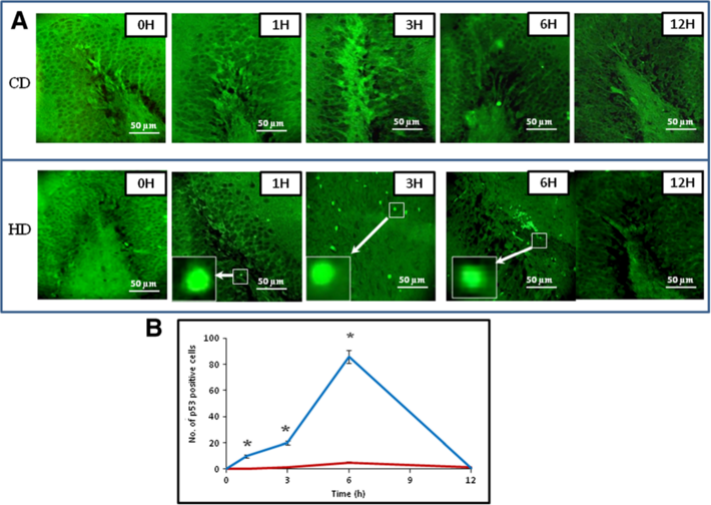
**Representative photographs showing HD-induced p53 activity in neuronal cells of the DG region. (A)** HD increased number of p53 positive cells in a time-dependent manner (Figs. HD, 1–6 H) during initial period (1–6 h) as compared to their respective CD groups (Figs. CD, 1–6 H). Enlarge view of p53 positive neuronal cells of the same figure (inset). Scale bar = 50 μM. **(B)** Quantitative analysis of p53 positive neuronal cells in DG region. Data are mean ± SEM of three independent experiments and analyzed by One-Way ANOVA followed by Tukey test. “*” Denote significantly different at p < 0.05 level. Blue line denote HD, red line denote CD.

Figure 2A shows the HD-induced p53 activity in neuronal cells. A time-dependent increased in the number of p53 positive cells were observed during initial period of HD (Figs. HD, 1–6 H) and then decreased 12 h post-HD. In contrast, except few cells (Figs. CD, 3 and 6 H), CD did not induce p53 activation as evidenced by p53 negative staining in neuronal cells at most of the time points studied. The quantitative analysis supports our results that HD increased number of p53 positive cells in a time-dependent manner (One-Way ANOVA; Figure 2B). The post hoc analysis (Tukey test) further suggests a significant increase of p53 positive neuronal cells during early period of HD (1–6 h).

### HD induces Bax protein expression

**Figure 3:**
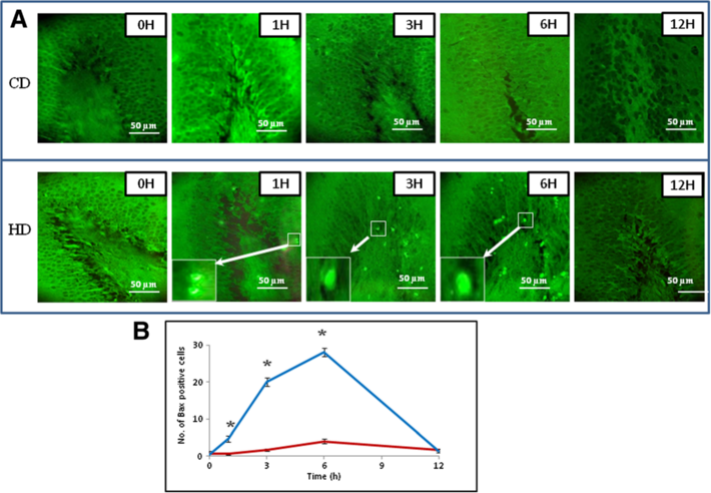
**Representative photographs showing HD-induced Bax protein expression in neuronal cells of the DG region. (A)** HD increased number of Bax positive cells in a time-dependent manner (Figs. HD, 1–6 H) during initial period (1–6 h) as compared to their respective CD groups (Figs. CD, 1–6 H). Enlarge view of Bax positive neuronal cells of the same figure (inset). Scale bar = 50 μM. **(B)** Quantitative analysis of Bax positive neuronal cells in DG region. Data are mean ± SEM of three independent experiments and analyzed by One-Way ANOVA followed by Tukey test. “*” Denote significantly different at p < 0.05 level. Blue line denote HD, red line denote CD.

Figure 3A shows the HD-induced Bax protein expression in neuronal cells. During initial period, HD increased number of Bax positive neuronal cells in a time-dependent manner (Figs. HD, 1–6 H). The number of Bax positive neuronal cells was declined drastically after 6 h post-HD. On the other hand, CD did not induce overexpression of Bax protein throughout the experimental period (Figs. CD, 1–6 H). The quantitative analysis supports our results that HD increased number of Bax positive cells in a time-dependent manner (One-Way ANOVA; Figure 3B). The post hoc analysis (Tukey test) further suggests a significant increase of Bax positive neuronal cells during early period of HD (1–6 h).

### HD increases cytochrome c concentration

**Figure 4:**
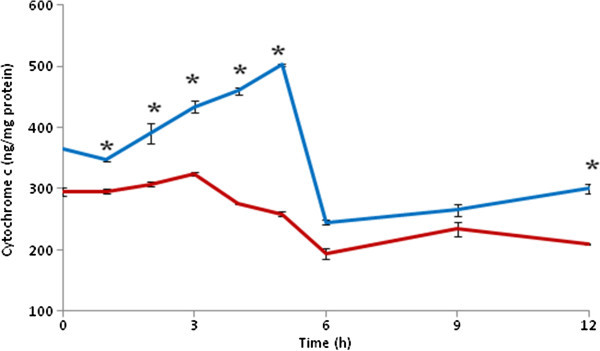
**HD increased cytochrome c concentration in brain.** Data are mean ± SEM of three independent experiments and analyzed by Two-Way ANOVA followed by Tukey test. “*” Denote significantly different at p < 0.05 level. Blue line denote HD, red line denote CD.

The quantitative analysis of cytochrome c suggest that HD significantly (p < 0.001) increased cytochrome c concentration throughout the experimental period (Two-Way ANOVA) as compare to CD (Figure 4). Post-hoc (Tukey test) analysis further revealed that cytochrome c concentration was significantly increased as early as 0 h after HD (366 ± 0.577 ng/mg protein) as compare to CD (295 ± 6.06 ng/mg protein) and found maximum after 5 h of HD (503 ± 1.732 ng/mg protein). Thereafter, cytochrome c concentration was comparable to CD group. On the other hand, cytochrome c concentration did not show any significant change in CD group at all the time points studied (Figure 4).

### HD increases caspases activity

**Figure 5:**
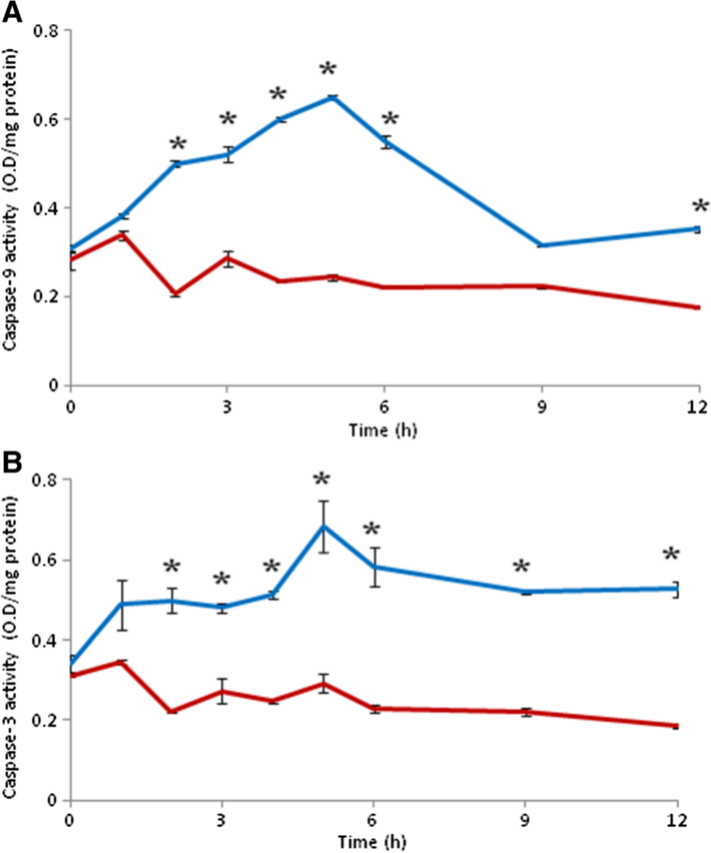
**HD increases caspase-9 (A) and caspase-3 (B) activities in brain.** Data are mean ± SEM of three independent experiments and analyzed by Two-Way ANOVA followed by Tukey test. “*” Denote significantly different at p < 0.05 level. Blue line denote HD, red line denote CD.

As shown in Figure 5, HD significantly (p < 0.001) increased both caspase-9 (Figure 5A) and as well as caspase-3 activities (Two-Way ANOVA) as compared to CD (Figure 5B). A time-dependent (One-Way ANOVA) increase of caspase-9 and caspase-3 activities were observed during early period of HD (0–5 h) followed by gradual decrease (Figure 5A and B) after 5 h of HD. Although caspase-9 activity was comparable to CD group after 12 h of HD (Figure 5A), caspase-3 activity was still high after 12 h of HD as compare to their respective CD groups (Figure 5B).

### HD induces DNA fragmentation

**Figure 6:**
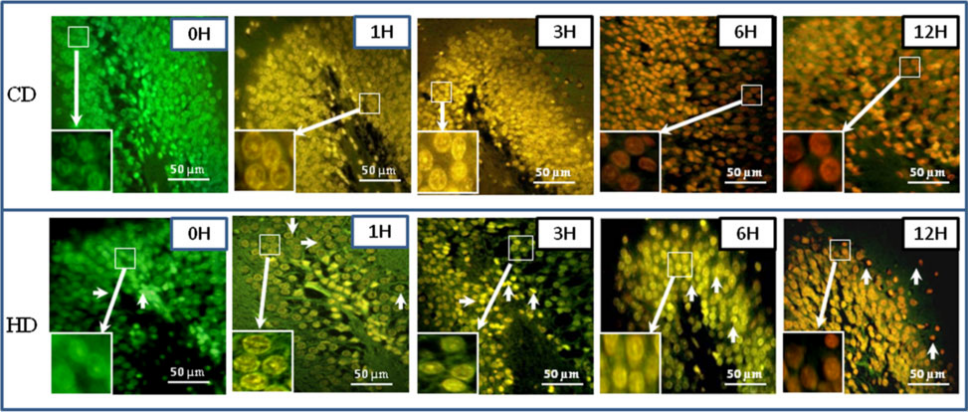
**Representative photographs showing HD induced apoptosis/necrosis in neuronal cells of the DG region of hippocampus.** Initiation of apoptosis was observed after 1 h (Fig. HD, 1 H) as evidenced by greenish yellow color fluorescence, apoptosis after 3 h as evidenced by yellowish orange color fluorescence, which was found maximum after 6 h post- HD (Fig. HD, 6H; arrows) followed by necrosis as evidenced by orange-red or reddish color fluorescence (Fig. HD, 12 H; arrows). CD induced necrosis as early as 3 h and continued till the end of the experimental period (Figs. CD, 3-12H) as evidenced by orange-red or reddish color fluorescence. Enlarge view of apoptotic or necrotic changes in neuronal cells of the same figure (inset). Scale bar = 50 μM.

As shown in Figure 6, HD induced apoptosis in the neuronal cells during early period followed by necrosis during later stages as evidenced by AO/EB staining. In the beginning, neuronal cells of CD as well as HD groups did not show any DNA fragmentation as evidenced by AO positive staining as a background colour fixed neuronal cells of both groups (Figs. CD and HD, 0 H). The early apoptotic cells were observed as early as 1 h post-HD as evidenced by greenish yellow color (Fig. HD 1-3H), which was gradually increased and found maximum after 6 h post-HD as evidenced by yellowish orange colour fluorescence (Fig. HD 6 H). The necrosis were seen only after 12 h post HD as most of the neuronal cells showed either orange-red or reddish colour fluorescence (Fig. HD,12 H). On the other hand, CD induced necrosis as early as 3 h post CD (Fig. CD, 3 H) and continued till the end of the experimental period (Fig. CD, 12 H).

## Discussion

In this study, we have shown that HD induces p53 activation and thereby mitochondria-caspase-mediated pathway is involved during HD-induced neuronal cell apoptosis in DG region of hippocampus. Although hanging is one of the most common approaches to commit suicide in human worldwide (Emet et al. 2010), the underlying mechanism during HD-induced neuronal cell apoptosis remains to be elucidated. We recently reported that HD induces generation of reactive oxygen species (ROS) and neuronal cell apoptosis in the DG region of rat brain (Khatun et al. 2013). Based on these studies, we proposed that the HD-induced generation of hydrogen peroxide may trigger neuronal cell apoptosis through the activation of p53 and mitochondria-mediated pathway. This hypothesis was strengthened by the observations of the present study that HD induced neuronal cell shrinkage and reduced total number of cells present in the DG region during initial experimental period (1–6 h). These data further strengthen our recent observations that HD induces shrinkage in DG region of the hippocampus in rat brain (Khatun et al. 2013). The similar structural changes in the neuronal cells after global brain ischemia produced by other methods have also been reported in several experimental animals (Oechmichen and Meissner 2006; Zheng et al. 2010).

The permanent global brain ischemia generated either by HD or by other methods induces generation of ROS and thereby morphological apoptotic changes in neuronal cells of DG region (Hyslop et al. 1995; Khatun et al. 2013; Chan 2001; Cao et al. 2011). The increased level of ROS acts as upstream signal to activate p53 and thereby neuronal cell apoptosis (Yu et al. 1999; Chan 2005; Endo et al. 2006; Kunimatsu et al. 2011). The p53 plays a central role in induction of apoptosis in wide variety of cells including neuronal cells (Endo et al. 2006; Mihara et al. 2003; Cao et al. 2001; Sugawara et al. 1999; Hong et al. 2010). Based on these studies, we hypothesize that increased level of ROS may induce p53 activity in neuronal cells undergoing HD-induced apoptosis. This hypothesis is further supported by our results that HD increased number of p53 positive neuronal cells during early period (1–6 h) as compare to their respective CD groups. Although role of p53 during HD-induced neuronal cell apoptosis has not been reported so far, global brain ischemia generated by other methods induced p53 activation and thereby neuronal cell apoptosis (Endo et al. 2006). The p53 activation in the cytoplasm may induce Bax protein expression as well as release of cytochrome c from mitochondria that initiate apoptotic signals in wide variety of cells (Miyashita et al. 1994; Selvakumaran et al. 1994; Zhan et al. 1994; Miyashita and Reed 1995; Brady et al. 1996; Xiang et al. 1998; Taylor et al. 2004; Vempati et al. 2007). In the present study, HD increased number of Bax positive neuronal cells and cytochrome c concentration in a time-dependent manner during initial period (1–6 h) as compare to their respective CD groups. Similarly, an increase of cytochrome c concentration and neuronal cell apoptosis have been reported during global brain ischemia induced by other methods (Mouw et al. 2002; Krajewski et al. 1999; Plesnila 2004). Taken together, these data suggest that HD induced p53 activation, overexpression of Bax protein and increased cytochrome c concentration during HD-induced neuronal cell apoptosis.

The release of cytochrome c from mitochondria activates upstream and downstream caspases to induce apoptotic cell death. Data of the present study revealed that HD induced both caspases-9 and caspases-3 activities during initial period of HD (1–6 h) as compared to their respective CD groups and then activity declined to basal level after 12 h of HD. These data further reconfirm our recent observations that HD increased caspases activity and thereby neuronal cell apoptosis (Gohel et al. 1999). Although substrates for caspases-7 and caspases-3 are not entirely specific and have some similar affinity, we analysed caspases-9 and caspase-3 activities in the present study. Caspase-3 is responsible for destruction of structural and specific proteins that leads to DNA damage and apoptotic cell death (Teschendorf et al. 2008; Han et al. 2011).

The DNA fragmentation in multiples of 180–200 base-pair is a hallmark feature of apoptosis and can be detected on the basis of color changes using nucleic acid-binding dye mix of AO/EB (Priyadarsini et al. 2010) in a fixed cell (Natesan et al. 2006). Normal cells with intact DNA show green fluorescence of AO. The extent of EB binding to fragmented DNA changes the color from green to yellow (for early apoptotic cells), orange (late apoptotic cells) and bright red color indicate necrotic cells (Gohel et al. 1999; Natesan et al. 2006; Priyadarsini et al. 2010). Data of the present study suggest that HD induced neuronal cell apoptosis during initial period of HD (1–6 H) as evidenced by yellow or yellowish orange color fluorescence in most of the neuronal cells in DG region. On the other hand, CD induced necrosis in neuronal cells as early as 3 h and continued till the end of experimental period (12 h post-CD). These data reconfirms our recent observations that the HD induces neuronal cell apoptosis in DG region of the rat brain (Khatun et al. 2013). Similarly, global brain ischemia produced by other methods has been reported to induce DNA fragmentation (MacManus et al. 1993; Han et al. 2011; Sharma and Gupta 2007).

## Conclusion

Data of present study suggest that HD induced p53 activation and thereby Bax protein expression during initial period (1–6 h) of HD. The over expression of Bax might have modulated mitochondria membrane potential and thereby cytochrome c release. A rise of cytochrome c concentration was associated with increased caspases activity and thereby DNA fragmentation, while CD induced necrosis in neuronal cells. These data suggest that p53 activation and thereby mitochondria-caspase-mediated pathway are involved during HD-induced neuronal cell apoptosis. Further studies are required using clinical samples to develop a cellular marker for the identification of exact timing of HD to address such clinically important forensic science issue.

## References

[CR1] Akdemir G, Ergungor F (1994). Global cerebral ischemic findings in a woman who attempted to commit suicide by hanging. Gen Physiol Biophys.

[CR2] Boghossian E, Clément R, Redpath M, Sauvageau A (2010). Respiratory, Circulatory, and Neurological Responses to Hanging: a review of animal models. J Forensic Sci.

[CR3] Brady HJ, Solomons GS, Bobel dijk RC, Berns AJ (1996). T-cells from Bax-alpha transgenic mice show accelerated apoptosis in response to stimuli but do not show restored DNA damage induced cell death in the absence of p53. J EMBO.

[CR4] Cao G, Minami M, Pei W, Yan C, Chen D, O’Horo C (2001). Intracellular Bax translocation after transient cerebral ischemia: implications for a role of the mitochondrial apoptotic signalling pathway in ischemic neuronal death. J Cereb Blood Flow Metab.

[CR5] Cao Y, Mao X, Sun C, Zheng P, Gao J, Wang X, Min D, Sun H, Xie N, Cai J (2011). Baicalin attenuates global cerebral ischemia/reperfusion injury in gerbils via anti-oxidative and anti-apoptotic pathways. Brain Res Bull.

[CR6] Chan PH (2001). Reactive oxygen radicals in signalling and damage in the ischemic Brain. J Cereb Blood Flow Metab.

[CR7] Chan PH (2004). Mitochondria and neuronal death/survival signaling pathways in cerebral ischemia. Neurochem Res.

[CR8] Chan PH (2005). Mitochondrial dysfunction and oxidative stress as determinants of cell death/survival in stroke. Ann N Y Acad Sci.

[CR9] Emet M, Saritas A, Aslan S, Uzkeser M, Cakr ZG, Coskun S (2010). Cervical spinal injury and hyoid fracture in a near-hanging victim. Hong Kong J Emerg Med.

[CR10] Endo H, Kamada H, Nito C, Nishi T, Chan PH (2006). Mitochondrial translocation of p53 mediates release of cytochrome *c* and hippocampal CA1 neuronal death after transient global cerebral ischemia in rats. J Neurosci.

[CR11] Ferrer I, Friguls B, Dalfo E, Justicia C, Planas AM (2003). Caspase-dependent and caspase-independent signalling of apoptosis in the penumbra following middle cerebral artery occlusion in the adult rat. Neuropathol Appl Neurobiol.

[CR12] Gohel A, McCarthy MB, Gronowicz G (1999). Estrogen prevents glucocorticoid-induced apoptosis in osteoblasts in vivo and in vitro. Endocrinol.

[CR13] Han B, Wang Q, Cui G, Shen X, Zhu Z (2011). Post-treatment of Bax-inhibiting peptide reduces neuronal death and behavioural deficits following global cerebral ischemia. Neurochem Intl.

[CR14] Hong LZ, Zhao XY, Zhang HL (2010). p53-mediated neuronal cell death in ischemic brain injury. Neurosci Bull.

[CR15] Hyslop PA, Zhang Z, Pearson DV, Phebus LA (1995). Measurement of striatal H_2_O_2_ by microdialysis following global forebrain ischemia and reperfusion in the rat: correlation with the cytotoxic potential of H_2_O_2_ in vitro. Brain Res.

[CR16] Jung Y, Hong S (2004). The effect of transient global ischemia on the rat dentate gyrus: apoptosis in the granular zone and neurogenesis in the subgranular zone. Korean J Anat.

[CR17] Khatun S, Chaube SK, Bhattacharya CN (2013). Generation of hydrogen peroxide mediates hanging death-induced neuronal cell apoptosis in the dentate gyrus of the rat brain. Brain Res Bull.

[CR18] Krajewski S, Krajewska M, Ellerby LM, Welsh K, Xie Z (1999). Release of caspase-9 from mitochondria during neuronal apoptosis and cerebral ischemia. Proc Natl Acad Sci.

[CR19] Kunimatsu T, Kobayashi K, Yamashita A, Yamamoto T, Lee MC (2011). Cerebral reactive oxygen species assessed by electron spin resonance spectroscopy in the initial stage of ischemia-reperfusion are not associated with hypothermic neuroprotection. J Clin Neurosci.

[CR20] Lebesgue D, Chevaleyre V, Zukin RS, Etgen AM (2009). Estradiol rescues neurons from global ischemia-induced cell death: multiple cellular pathways of neuroprotection. Steroids.

[CR21] MacManus JP, Buchan AM, Hill IE, Rasquinha I, Preston E (1993). Global ischemia can cause DNA fragmentation indicative of apoptosis in rat brain. Neurosci Lett.

[CR22] Mihara M, Erster S, Zaika A, Petrenko O, Chittenden T, Pancoska P, Moll UM (2003). p53 has a direct apoptogenic role at the mitochondria. Mol Cell.

[CR23] Miyashita T, Reed JC (1995). Tumor suppressor p53 is a direct transcriptional activator of the human Bax gene. Cell.

[CR24] Miyashita TS, Krajewski S, Krajewska M, Wang HG, Lin HK, Liebermann DK (1994). Tumour suppressor p53 is a regulator of bcl-2 and Bax gene expression in vitro and in vivo. Oncogene.

[CR25] Mouw G, Zechel JL, Zhou Y, Lust WD, Selman WR, Ratcheson RA (2002). Caspase-9 inhibition after focal cerebral ischemiaimproves outcome following reversible focal ischemia. Metab Brain Dis.

[CR26] Namura S, Zhu J, Fink K, Endres M, Srinivasan A, Tomaselli KJ (1998). Activation and cleavage of caspase-3 in apoptosis induced by experimental cerebral ischemia. J Neurosci.

[CR27] Natesan S, Kataria JM, Dhama K, Bhardwaj N (2006). Anti-neoplastic effect of chicken anemia virus VP3 protein (apoptin) in rous sarcoma virus-induced tumours in chicken. J Gen Virol.

[CR28] Oechmichen M, Meissner C (2006). Cerebral hypoxia and ischemia: the forensic point of view. J Forensic Sci.

[CR29] Plesnila N (2004). Role of mitochondrial proteins for neuronal cell death after focal cerebral ischemia. Acta Neurochir Suppl.

[CR30] Priyadarsini RV, Murugan RS, Maitreyi S, Ramalingam K, Karunagaran D, Nagini S (2010). The flavonoid quercetin induces cell cycle arrest and mitochondria-mediated apoptosis in human cervical cancer (HeLa) cells through p53 induction and NF-κB inhibition. Euro J Pharmacol.

[CR31] Selvakumaran M, Lin HK, Miyashita T, Wang HG, Krajewski S, Reed JC (1994). Immediately early upregulation of Bax expression by p53 but not TGF-1: a paradigm for distinct apoptotic pathways. Oncogene.

[CR32] Sharma SS, Gupta S (2007). Neuroprotective effect of MnTMPyP, a superoxide dismutase/catalase mimetic in global cerebral ischemia is mediated through reduction of oxidative stress and DNA fragmentation. Eur J Pharmacol.

[CR33] Sugawara T, Fujimura M, Morita-Fujimura Y, Kawase M, Chan PH (1999). Mitochondrial release of cytochrome c corresponds to the selective vulnerability of hippocampal CA1 neurons in rats after transient forebrain ischemia. J Neurosci.

[CR34] Sugawara T, Noshita N, Lewen A, Gasche Y, Ferrand-Drake M, Fujimura M (2002). Overexpression of copper/zinc superoxide dismutase in transgenic rats protects vulnerable neurons against ischemic damage by blocking the mitochondrial pathway of caspase activation. J Neurosci.

[CR35] Sugawara T, Fujimura M, Noshita N, Kim GW, Saito A, Hayashi T (2004). Neuronal death/survival signalling pathways in cerebral ischemia. NeuroRx.

[CR36] Taylor SL, Weng SL, Fox P, Duran EH, Morshedi MS, Oehninger S (2004). Somatic cell apoptosis markers and pathways in human ejaculated sperm: potential utility as indicators of sperm quality. Mol Human Repro.

[CR37] Teschendorf P, Padosch SA, Spohr F, Albertsmeier M, Schneider A, Vogel P (2008). Time course of caspase activation in selectively vulnerable brain areas following global cerebral ischemia due to cardiac arrest in rats. Neurosci Lett.

[CR38] Traystman RJ (2003). Animal models of focal and global cerebral ischemia. J ILAR.

[CR39] Vempati UD, Diaz F, Barrientos A, Narisawa S, Mian AM, Millán JL (2007). Role of Cytochrome *c* in apoptosis: increased sensitivity to tumor necrosis factor alpha is associated with respiratory defects but not with lack of cytochrome *c* release. Mol Cell Biol.

[CR40] Xiang H, Kinoshita Y, Knudson CM, Korsmeyer SJ, Schwartzkroin PA, Morrison RS (1998). Bax involvement in p53-mediated neuronal cell death. J Neurosci.

[CR41] Yamasaki S, Kobayashi AK, Nishi K (2007). Evaluation of Suicide by Hanging. Forensic Sci Med Pathol.

[CR42] Yu J, Zhang L, Hwang PM, Rago C, Kinzler KW, Vogelstein B (1999). Identification and classification of p53-regulated genes. Proc Natl Acad Sci.

[CR43] Zhan Q, Fan S, Bae I, Guillof C, Liebermann DA, O’Conner PM (1994). Induction of Bax by genotoxic stress in human cells correlates with normal p53 status and apoptosis. Oncogene.

[CR44] Zhang GQ, Zhou B, Du B, Yang ZH, Zhang BL, Zhu YH (2006). Expression of HIF1-alpha on myocardium and lung in rats model of asphyxia death. [In Chinese]. Fa Yi Xue Za Zhi.

[CR45] Zheng W, Honmou O, Miyata K, Harada K, Suzuki J, Liu H (2010). Therapeutic benefits of human mesenchymal stem cells derived from bone marrow after global cerebral ischemia. Brain Res.

